# Genetic susceptibility of bladder cancer in the Lebanese population

**DOI:** 10.1186/s12920-022-01372-z

**Published:** 2022-10-17

**Authors:** Hampig Raphael Kourie, Bahaa Succar, Eliane Chouery, Cybel Mehawej, Nizar Ahmadieh, Joseph Zouein, Avedis Mardirossian, Nadine Jalkh, Ghassan Sleilaty, Joseph Kattan, Elie Nemr

**Affiliations:** 1 Hematology-Oncology Department, Faculty of Medicine, Saint Joseph University of Beirut, Beirut, Lebanon; 2 Medical Genetics Unit, Faculty of Medicine, Saint Joseph University, Beirut, Lebanon; 3 Department of Human Genetics, Gilbert and Rose-Marie Chagoury School of Medicine, Lebanese American University, Byblos, Lebanon; 4 Urology Department, Faculty of Medicine, Saint Joseph University of Beirut, Beirut, Lebanon

**Keywords:** Bladder cancer, Genetic susceptibility, Genetic variant, Gene, Lebanese

## Abstract

**Background:**

Bladder cancer (BC) is the 10^th^ most frequent tumor worldwide. Evidence shows an association between elevated risk of BC and various single nucleotide polymorphisms (SNP). BC incidence was the highest in Lebanon according to Globocan 2018 report, but little is known about the genetic susceptibility of Lebanese people to this disease. We aim to evaluate whether this prominent incidence of BC in Lebanon is attributable to known coding genetic variants.

**Methods:**

A case-control study was conducted at Hotel-Dieu de France Hospital, Beirut. A cohort of 51 Lebanese patients with BC were recruited between 2017 and 2020. Whole Exome Sequencing (WES) was performed on peripheral blood samples to detect coding genetic variants in the patients. An in-house database including WES data from 472 Lebanese individuals served as control. Literature review of the genetic predisposition to BC was conducted to establish a database of variants known to influence the risk of BC. In-common SNPs were identified between cases and the aforecited database, and their allelic frequencies was quantified in the former and in controls. Comparative analysis of the allelic frequencies of each in-common SNP was carried out between cases, controls, and the genome aggregation database (gnomAD). Analysis was performed by applying the binomial law and setting the p-value to 10^− 10^.

**Results:**

484 polymorphisms associated with BC were extracted from the literature review ;151 of which were in-common with the 206 939 variations detected by WES in our cases. Statistically significant differences (p-value < 10^− 10^) in allelic frequencies was seen in 11 of the 151 in-common SNPs, but none of which corresponds with a higher BC risk. Moreover, rs4986782 variant in the *NAT1* gene is not associated with BC in the Lebanese population. `.

**Conclusion:**

This is the first next-generation sequencing (NGS)- based study investigating BC risk in a Lebanese cohort of 51 patients. The majority of known exonic variants in the literature were not associated with BC in our patients. Further studies with larger sample sizes are warranted to explore the association of BC in our population with known non-coding genetic variants, and the remainder of WES-generated private Lebanese variants.

**Supplementary Information:**

The online version contains supplementary material available at 10.1186/s12920-022-01372-z.

## Background

Bladder cancer (BC) is the most frequent malignant tumor of the urinary tract and the 10^th^ most frequent cancer worldwide with an annual incidence surpassing half a million new cases in 2018 (424 000 in males and 125 000 in females) from all ages. In terms of mortality, it is ranked the 12^th^ worldwide with 200,000 deaths each year [1]. According to the United Nations, the world population is increasing and is projected to exceed 9.7 billion people by 2050. Thus, the global incidence rate of BC is expected to increase, placing a heavy burden on the healthcare system [1].

Environmental exposures, notably smoking [2], and genetic risk factors impact the incidence of BC [3, 4]. Owing to the advances in genomic analysis, several single center [4, 5] and multi-center case-control studies [6, 7],genome-wide association studies(GWAS) [6, 8–10], systematic reviews, and meta-analyses with large pooled samples have shown an elevated risk of BC with different SNPs (Single Nucleotide Polymorphisms) across multiple genetic pathways [11, 12]. Researchers have also identified polymorphisms in both coding and non-coding regions that influence the risk of BC [13, 14]. Additionally, a large body of evidence suggests the presence of population-related genetic polymorphisms that alter the susceptibility to BC [5, 6, 15, 16] such as rs9642880 in individuals of European ancestry [6], and rs10936599 in the Turkish population [5]. Other investigators observed inter-population differences regarding the effect of SNPs on BC, whereby the same genetic variation enhances susceptibility of BC in one population, but mitigates the risk in another group of people [12].*Song* and companions concluded that Asians have an increased risk of BC with rs699947, whereas Africans exhibit reduced risk of BC with the aforementioned SNP [12].

According to the Globocan 2018 report, Lebanon has the highest global incidence of BC in females and the 2^nd^ highest global incidence in males [17]. However, little is known about the genetic predisposition of the Lebanese people to BC. This underscores the need to investigate the influence of genetic risk factors of BC in a cohort of Lebanese patients.

Admittedly, this study aims to establish a comprehensive database of the variations associated with BC reported in the current literature (known variants) and to evaluate, by Whole Exome Sequencing (WES), whether the high BC incidence in a Lebanese cohort is attributable to a known coding genetic variant. To our knowledge, there are no previous investigations using next generation sequencing (NGS) technology to explore the genetic predisposition of Lebanese people to BC. We hypothesize that novel polymorphisms specific to the Lebanese population contribute to the highly reported incidence of BC in Lebanon.

## Materials and methods

### Literature search strategy

**Fig. 1:**
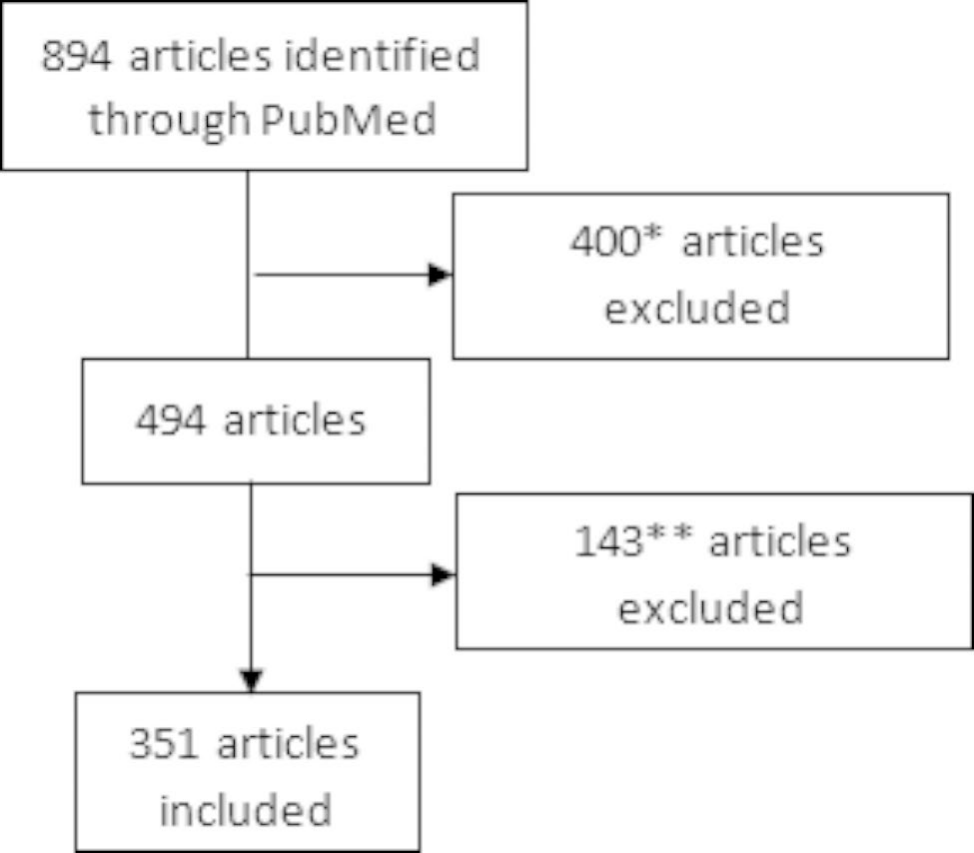
Flow Diagram detailing articles’ selection steps *400 *Articles unrelated to the genetic susceptibility of BC are eliminated* ** 143 *Articles excluded: Hereditary Bladder cancer studies, Epigenetic studies, Haplotype analysis*

The literature search was conducted on PubMed using the following keywords “Bladder Cancer”, “Genetic predisposition», and “Genetic susceptibility” to collect the articles related to BC susceptibility and genetic association studies between January 2000 and October 2020. The last search was carried out on 10/10/2020 and generated 894 results. Initially, all articles exploring genetic risks for BC between January 2000 and October 2020 were included and all projects unrelated to this subject that appeared in the search results were eliminated after checking each paper manually. After reviewing each article, the papers that targeted hereditary BC, epigenetic, haplotype studies, or various cancer types without emphasizing BC were excluded. The selection process is illustrated in Fig. 1.

### Lebanese cohort

The studied sample consists of 51 patients with BC from different Lebanese areas, who presented to Hotel-Dieu de France Hospital in Beirut, Lebanon, a tertiary university hospital, between January 2017 and June 2020.

All methods were carried out in accordance with the declaration of Helsinki and performed in accordance with relevant guidelines and regulations. The project was approved by the Ethics Committee of Saint Joseph University, Beirut, Lebanon (Approval number : Tfem-2020-85). All patients signed an informed consent for participation, sample collection, and data publication. Peripheral blood was then collected from each individual enrolled in this study and DNA was extracted using the salting-out method [18].

### WES analysis

**Exon capture and sequencing**: The exome was captured using the SureSelect Human All Exons, reagents (Agilent Inc.® Santa Clara, CA) according to the manufacturer’s standard protocol. The concentration of each library was determined using Agilent’s QPCR NGS Library Quantification Kit (G4880A). Samples were pooled prior to sequencing with a final concentration of each sample equal to 10 nM. Sequencing was performed on the Illumina HiSeq2000 platform using TruSeq v3 chemistry.

**Mapping and alignment**: Reads files (FASTQ) were generated from the sequencing platform via the manufacturer’s proprietary software. Reads were aligned to the hg19/b37 reference genome using the Burrows-Wheeler Aligner (BWA) package v0.6.1 [19]. Local realignment of the mapped reads around potential insertion/deletion (Indel) sites was carried out with the Genome Analysis Tool Kit (GATK) v1.6 [20]. Duplicate reads were marked using Picard v1.62. Additional BAM file manipulations were performed with Samtools 0.1.18 [21]. Base quality (Phred scale) scores were recalibrated using GATK’s covariance recalibration. SNP and Indel variants are called using the GATK Unified Genotyper for each sample [22]. Variants were called using high stringency settings and annotated with VarAFT software 2.131 [23] containing information from dbSNP147 and GnomAD [https://gnomad.broadinstitute.org]. Finally, bioinformatics analysis of the data was carried out and focused on all the variations detected in the cohort of 51 Lebanese patients that were in common with those included in the list established by our literature review.

### Statistical analysis

The allelic frequencies of the variations were calculated in our cohort of 51 Lebanese patients with BC. The frequencies of these variations in the local Lebanese database (sample control of 472 Lebanese individuals) and the international population database (according to the GnomAD database) have been added to Additional file 1. Comparative analysis of the three frequencies for each selected SNP was carried out by applying the binomial law and setting the p-value to 10^− 10^.

## Results

### Literature review and association studies

The results of the literature study were grouped into one table (More explicit details can be found in Additional file 2) that includes all the cited polymorphisms: those that are associated with an increased risk for BC, those that present a protective effect, and finally the SNPs that have conflicting associations with BC.

Our search strategy generated 894 records from which 351 articles as shown in Fig. 1. Overall, 484 variants in 269 genes were considered as presenting a positive association with BC. A detailed list of all selected variants with their corresponding references is provided in Additional file 2. Table 1 includes a summary of the types and locations of the selected 484 variations.

**Table 1:** The different types and localizations of the 484 known variants extracted from the literature review

Identified SNPs	N = 484
Nc transcript variant	1
Intergenic variants	30
Non-Coding Transcript Variants	18
14-bp insertion/deletion	1
Upstream gene variants	73
Downstream gene variants	18
5’ Prime UTR Variants	3
3’ Prime UTR Variants	36
Intron Variants	167
Synonymous Variants	20
Missense Variants	108
Stop Gained Variants	3
Frameshift Variants	2
Inframe Deletion	1
Splice Acceptor Variants	2
Initiator Codon Variant	1

*SNP: single nucleotide polymorphism N: number of variants UTR: untranslated region*

### Characteristics of the included clinical samples

The studied population consists of 51 Lebanese patients including 9 females and 42 males, from various and different areas across Lebanon. The mean age at BC diagnosis is 67 years in both genders with 68.5 in males and 59.5 in females.

### Polymorphisms detected in our cohort of 51 lebanese patients

WES performed in the 51 patients with BC generated a total of 206 939 variations. Among the variants detected in our cohort, a total of 151 were found to be present in the list of variants known to be associated with BC. Those were designated as “in common variations” (More details can be found in Additional file 1). The frequency of each selected SNP was evaluated in our cohort and compared to that in our in-house Lebanese database of 472 controls as well as in the Genome Aggregation Database (GnomAD).

These variations are briefly summarized in Table 2.

**Table 2:** List of the different types and localizations of the 151 identified “in common variations”

Identified SNPs	N = 151
Missense Variants	93
Synonymous Variants	19
Stop Gained Variant	1
Frameshift Variants	2
Inframe Deletion	1
Splice Acceptor Variants	2
Intron Variants	7
3 Prime UTR Variants	13
5 Prime UTR Variants	2
Non Coding Transcript Variants	8
upstream_gene_variants	3

*SNP: single nucleotide polymorphisms N: number of variants UTR: untranslated region*

In the literature study, 167 (34.5%) variations were intronic, whereas our study enabled the detection of only 7 intronic variations, representing 4.6% of the 151 selected “in common SNPs”. As for the exonic variants, 134 exonic variations were previously reported to be associated with BC risk; of these, 116 variations were detected by WES in our study. Therefore, 86.6% of these exonic variations were covered by WES in our sample.

A thorough analysis of the 151 in common SNPs led to the selection of 11 candidate SNPs based on the difference between their frequencies in the studied Lebanese cohort (A) compared to the local Lebanese database (B) and/or to the GnomAD database (C) (Table 3). Of these, 5 are pyrimidine to pyrimidine changes. Those 11 SNP were grouped into 3 different categories:

**Table 3:** The 11 candidate SNPs showing significant frequencies differences between the different evaluated populations

Groups	Types of SNPs	Type of amino acid change	Variants	Genes	A= Frequency in the studied Lebanese cohort	B= Frequency in the local Lebanese database	C= Frequency in gnomAD	Difference A vs. B	Difference A vs. C	99.9999999% CI of A
Group 1	3 Prime UTR Variant	Pyrimidine to pyrimidine	rs1707	*HLA-G*	0.529411765	0.20127119	0.8574	**Yes**	**Yes**	[0.24714813–0.798697334]
	3 Prime UTR Variant	Pyrimidine to purine	rs1063320	*HLA-G*	0.215686275	0.0286017	0.516	**Yes**	**Yes**	[0.04591686–0.509931283]
Group 2	3 Prime UTR Variant	Pyrimidine to pyrimidine	rs9299	*HOXB-AS3*	0.12745098	0.19597458	0.531	No	**Yes**	[0.01361905–0.402416044]
	3 Prime UTR Variant	Pyrimidine to pyrimidine	rs696	*NFKBIA*	0.019607843	0.14830509	0.4574	No	**Yes**	[3.04E-06-0.227119758]
	Non-Coding Transcript Variant	Purine to purine	rs1061217	*SLAMF1*	0.509803922	0.28495763	0.00003191	No	**Yes**	[0.23147854–0.783803061]
	Intron Variant	Pyrimidine to pyrimidine	rs1799794	*XRCC3*	0.009803922	0.00423729	0.2219	No	**Yes**	[7.4861E-09-0.203334752]
	Intron Variant	Pyrimidine to pyrimidine	rs1799796	*KLC1; XRCC3*	0.205882353	0.194915254	0	No	**Yes**	[0.04162045–0.49880505]
	Intron Variant	Purine to purine	rs1805335	*RAD23B*	0.607843137	0.44809322	0.000004051	No	**Yes**	[0.313778362–0.854357768]
Group 3	3 Prime UTR Variant	Purine to pyrimidine	rs1710	*HLA-G*	0.431372549	0.150423729	0.5169	**Yes**	No	[0.172681469–0.720337798]
	Upstream gene_variant	Not applicable	rs3213239	*PINLYP*	0.411764706	0.13559322	0.6146	**Yes**	No	[0.158961335–0.703483575]
	Intron Variant	Purine to pyrimidine	rs1801320	*RAD51*	0.019607843	0	0.1266	**Yes**	No	[3.03998E-06-0.227119758]

CI: confidence interval p-value :10^–10^. In “Difference A vs. B” and “Difference A vs. C” columns, “Yes” designates a statistically significant difference, while “No” indicates that there is no statistically significant difference

- Group 1: 2 SNPs (rs1707 and rs1063320) showed a statistically significant difference between the cohort of 51 patients (A) and the sample of 472 controls (B) on one hand, and between (A) and the global database / gnomAD (C) on the other hand.
- Group 2: 6 SNPs (rs9299, rs696, rs1061217, rs1799794, rs1799796, rs1805335) showed only a statistically significant difference between the cohort of 51 patients (A) and the global database / gnomAD (C) but did not show any significant difference with the local database.
- Group 3: 3 SNPs (rs1710, rs3213239, rs1801320) showed only a statistically significant difference between the cohort of 51 patients (A) and the sample of 472 Lebanese controls (B) but did not show significant differences with the international database.

### Evaluation of the candidate SNPs in the NAT1 gene

We compared our results with those obtained by association studies in the Lebanese population published in 2012 and 2013 [28]-[29] which evaluated the following *NAT1* variants: rs15561, rs1057126, and rs4986782. Our analysis focused on one variant, the exonic SNP “rs4986782” in *NAT1*, that is covered by the technique used in our study which is WES. Our findings show a lack of association between the rs4986782 and BC in our Lebanese cohort.

## Discussion

BC is the most common malignant tumor of the urinary system. It can be present as an isolated form or as a part of a cancer syndrome. Gene sequencing and GWAS have identified in recent decades several new candidate genes and regions that might modulate BC risk. However, the contribution of genetic factors remains unclear in lots of cases, thus complicating the approach of genetic counseling in these patients and rendering our understanding of the molecular mechanisms driving BC limited to date.

In this project, we focused, initially, on reviewing the literature related to the genetic variants associated with BC. This allowed us to establish a list of 484 polymorphisms located in 269 genes, known to be variants associated with BC. By gathering and summarizing this information, our work provides a comprehensive review related to the genetic predisposition to BC. Our review also highlighted the differences in the association of specific SNPs and BC between populations. It is indeed noted that the same SNP is associated with an increased risk of BC in one population while it is protective or not associated with BC in another population. For example, the *TP53* gene’s variant rs1042522 is not associated with BC in the Spanish population [26] while it increases the BC risk in the Bangladeshi population [27] and it decreases this risk in the Brazilian population [28]. The *APOBEC3A* gene’s variant rs1014971 is associated with an increased risk of BC in the Japanese [29] and Swedish populations [30] but it is not associated with BC in the Taiwanese population [31]. *The MMP1* gene’s variant rs1799750 also increases the risk of BC in the Turkish [32] and North Indian populations [33] while it decreases it in the Polish population [34]. Those SNPs were considered as variants with conflicting interpretations.

We then conducted a next-generation sequencing (NGS)-based study on a cohort of 51 Lebanese patients affected with BC. This study allowed us to detect 206 939 SNPs in the studied population. Those were then filtered to select the variants that are included in the list of 484 SNPs known to be associated with BC; in total 151 SNPs were selected in our cohort of 51 Lebanese patients with BC. Subsequently, a thorough analysis was performed to determine candidate SNPs based on the difference in their frequencies between the studied cohort, the Lebanese population database, and the global international database. This subsequent analysis allowed us to narrow the list to 11 candidate SNPs with a possible association with BC.

The selected variants were then classified into three different groups: The first (group 1) includes 2 SNPs in the *HLA-G* gene (rs1707 and rs1063320) that showed a statistically significant difference between the cohort of 51 patients (A) and the sample of 472 controls (B) on one hand, and between (A) and the global database / gnomAD (C) on the other hand. However, while their frequency in our cohort was higher than that in the Lebanese database, it was lower than that in the international database, which suggests that their association with BC is less likely to be possible. The second group (group 2) includes 6 SNPs (rs9299, rs696, rs1061217, rs1799794, rs1799796, rs1805335) that showed only a statistically significant difference between the cohort of 51 patients (A) and the global database / gnomAD (C) but did not show any significant difference with the local database. Those are likely to be either polymorphisms that are “private” to the Lebanese population in general or variants that are rare in our population. Interestingly, among those 6 SNPs, two SNPs, the rs1061217 in *SLAMF1* and rs1805335 in *RAD23B* showed a high frequency in our cohort compared to the international database, and the Lebanese local database. However, this difference is statistically significant between our cohort and the international database, and not between the cohort and the Lebanese database. This suggests one of the following scenarios: either the cohort size is small and statistical significance between our cohort and the local database will be obtained with bigger sample size or those two variants are « private to the Lebanese population » without being associated with BC.

Last but not least, group 3 consists of 3 SNPs (rs1710, rs3213239, rs1801320) that showed only a statistically significant difference between the cohort of 51 patients (A) and the sample of 472 Lebanese controls (B) but showed a comparable frequency with that reported in international databases (as differences were not statistically significant), thus indicating they are less likely to be contributing to the disease.

Furthermore, our data was then evaluated based on association studies previously performed in the Lebanese population and published in 2012 and 2013 [28]-[29], where three variations in the *NAT1* gene, rs4986782, rs1057126, and rs15561 were reported to be associated with an increased risk for BC in the Lebanese population. Our study that is based on WES analysis thus focusing only on the coding SNP, the rs4986782, showed a lack of association between the latter and BC in the Lebanese population. The remaining variants located at the 3’UTR region of *NAT1* could not be evaluated by WES. On the other hand, the previous two studies compared the frequency of SNPs to a total of 105 controls individuals, while in our study, the SNPs were compared to a control sample of 472 individuals, considered to be more representative of the Lebanese population. Therefore, our results suggest that *NAT1* (rs4986782) is not associated with BC risk in the Lebanese population.

Altogether, the absence of relevant variants that show a statistically significant difference between our cohort, the Lebanese local database, and gnomAD can be due to the possibility that the variants responsible for the high frequency of BC in the Lebanese population were not covered by this study because our approach included only exonic and known SNPs. Another hypothesis would be that the limited size of our cohort did not enable us to reach a statistically significant difference between the frequency of relevant SNPs in our cohort compared to the in-house Lebanese database and the international database, as was mentioned in the case of the two SNPs the rs1061217 in *SLAMF1* and rs1805335 in *RAD23B*. Further studies are thus needed to complement our findings.

### Study limitations

WES technique has different limitations; it aims to detect variations mainly in exons and exon/intron junctions to study the acceptor and donor sites of canonical splicing but excludes introns and much of the non-coding 5’ and 3’ UTR regions. This suggests that our findings focused on exonic variants might have missed some intronic variants relevant to BC risk in the Lebanese population.

In conclusion, this project represents the first investigation by NGS of 51 Lebanese patients with BC. In contrast to the literature review, the majority of known exonic variants (86.6%) were not found to be associated with BC in the Lebanese population. Finally, despite the challenging interpretation of WES data, we aim to re-analyze all the obtained data related to the 51 patients with BC to investigate the possible presence of an association between private “Lebanese variants” and BC risk in this population and determine if novel « non-previously reported SNPs » increase BC in the Lebanese population.

On the other hand, one should not ignore the contribution of environmental factors to this type of cancer. Indeed, several studies have described the influence of certain environmental factors on genetic variants and the subsequent epigenetic modification of the risk of BC [35–38]. Many other studies have also shown the effect of intergenic interaction (regions regulatory genes, intergenic sequences, and introns) on BC risk [39, 40]. Therefore, further studies using Whole Genome Sequencing (WGS) or epigenome studies could be needed to explore these factors in all populations in general.

## Electronic supplementary material

Below is the link to the electronic supplementary material.

Supplementary Material 1

Supplementary Material 2

## Data Availability

The datasets used and/or analyzed during the current study are available from the corresponding author on reasonable request.

## List of abbreviations

BC: Bladder Cancer
GWAS: Genome-Wide Association Study
SNP: Single nucleotide polymorphism
BWA: Burrows-Wheeler Aligner
GATK: Genome Analysis Tool Kit
UTR: Untranslated Regions
GnomAD: Genome Aggregation Database
NGS: Next-generation sequencing
WES: Whole Exome Sequencing
WGS: Whole Genome Sequencing
